# Evaluation of the Machine Performance Check application for TrueBeam Linac

**DOI:** 10.1186/s13014-015-0381-0

**Published:** 2015-04-21

**Authors:** Alessandro Clivio, Eugenio Vanetti, Steven Rose, Giorgia Nicolini, Maria F Belosi, Luca Cozzi, Christof Baltes, Antonella Fogliata

**Affiliations:** IOSI, Oncology Institute of Southern Switzerland, Medical Physics Unit, Bellinzona, 6504 Switzerland; Varian Medical Systems Imaging Laboratory, Baden-Dättwil, Switzerland; Radiotherapy and Radiosurgery Department, Humanitas Research Hospital, Milan-Rozzano, Italy

**Keywords:** TrueBeam, Quality assurance, Machine performance

## Abstract

**Background:**

Machine Performance Check (MPC) is an application to verify geometry and beam performances of TrueBeam Linacs, through automated checks based on their kV-MV imaging systems. In this study, preliminary tests with MPC were analyzed using all photon beam energies of our TrueBeam, comparing whenever possible with external independent checks.

**Methods:**

Data acquisition comprises a series of 39 images (12 with kV and 27 with MV detector) acquired at predefined positions without and with the IsoCal phantom in the beam, and with particular MLC pattern settings. MPC performs geometric and dosimetric checks. The geometric checks intend to test the treatment isocenter size and its coincidence with imaging devices, the positioning accuracy of the imaging systems, the collimator, the gantry, the jaws, the MLC leaves and the couch position. The dosimetric checks: refer to a reference MV image and give the beam output, uniformity and center change relative to the reference. MPC data were acquired during 10 repetitions on different consecutive days.

Alternative independent checks were performed. Geometric: routine mechanical tests, Winston-Lutz test for treatment isocenter radius. Dosimetric: the 2D array StarCheck (PTW) was used just after the MPC data acquisition.

**Results:**

Results were analyzed for 6, 10, 15 MV flattened, and 6, 10 MV FFF beams. Geometric checks: treatment isocenter was between 0.31 ± 0.01 mm and 0.42 ± 0.02 mm with MPC, compared to 0.27 ± 0.01 mm averaged on all energies with the Winston-Lutz test. Coincidence of kV and MV imaging isocenters was within 0.36 ± 0.0 and 0.43 ± 0.06 mm, respectively (0.4 ± 0.1 mm with external tests). Positioning accuracy of MLC was within 0.5 mm; accuracy of jaws was 0.04 ± 0.02, 0.10 ± 0.05, −1.01 ± 0.03, 0.92 ± 0.04 mm for X1, *X*2, Y1, Y2 jaws, respectively, with MPC. Dosimetric tests: the output stability relative to the baseline was in average 0.15 ± 0.07% for MPC to compare with 0.3 ± 0.2% with the independent measurement.

**Conclusions:**

MPC proved to be a reliable, fast and easy to use method for checking the machine performances on both geometric and dosimetric aspects.

**Electronic supplementary material:**

The online version of this article (doi:10.1186/s13014-015-0381-0) contains supplementary material, which is available to authorized users.

## Background

The goal of all the quality assurance (QA) programs for linear accelerators is to guarantee that the machine characteristics do not deviate significantly from their baseline values acquired at the time of acceptance and commissioning [1]. Many publications describe procedures and conditions for testing, as for example the International Electrotechnical Commission (IEC) publications [2,3]. The main sections of a QA program can be categorized as: dosimetric, mechanical, imaging, special devices and procedure, safety. The AAPM Task Group 142 [1] was published in 2009 as an update and completion of the AAPM Task Group 40 [4] to give recommendation on all the machine parts, adding the newer ancillary delivery technologies (dynamic, intensity modulated IMRT, or stereotactical SRS/SBRT treatments) as well as the imaging devices that are nowadays an integral part of the Linac: X-ray imaging, photon portal imaging, cone-beam CT. In particular in this report 142 different tolerances have been recommended, according to the specific usage of the machine: non-IMRT, IMRT, SRS/SBRT. To report few specific recommended parameters: for IMRT machines the MLC leaf position accuracy and repeatability tolerance is ±1 mm and the MLC spoke shot ≤1 mm radius. The imaging system accuracy should be better than 2 mm for non SRS/SBRT machines, decreasing to 1 mm for Linacs used for stereotactical treatments. The dosimetric parameters (e.g. flatness and symmetry) should stay within ±1% from baseline.

The advent in the new Linacs of flattening filter free modes (FFF beams), not yet covered by the AAPM Report 142, having very high dose rates and bell-shaped lateral profiles increased their use for stereotactical treatment. Such profiles, so different in shape from the corresponding flattened one, faced to the need of evaluating profile parameters that cannot be identical to the standard flattened beam parameters, but should keep the same concepts and could be used in the same way as the analogous for standard fields [5].

The comprehensive modern Linac system, including the MV electronic portal imaging device, the kV on-board imager allowing also the acquisition of cone-beam CT, shall be checked in terms of coincidence of all the isocenters as part of the QA program. At the same time those ancillary devices are important instruments that could be used to check and evaluate the mutual isocenters’ positioning, as well as the constancy of the machine performances in terms of mechanical, collimating parameters, and also dosimetric constancy for all available beams, flattened or unflattened.

The concept of the coincidence of all the isocenters present in the system (mechanical isocenter, treatment beam isocenter, kV imaging system isocenter, and MV imaging system isocenter) has been deeply analyzed and there are recommendations concerning this subject. For example the AAPM Task Group 179 [6] gives strength to the ±1 mm tolerance that should be achieved when stereotactic treatments are in place. The same tolerance was suggested by Yoo et al. [7] for on-board imagers for stereotactical usage of the Linac. Considering that the usage of such treatments is rapidly increasing in the last years due to clinical reasons, it is becoming more and more important to make easily available instruments to apply on regular basis and included in the QA program, able to fastly evaluate the Linac performances in terms of accuracy of its main parameters, geometrical, but also dosimetric for what concerns the beam stability and constancy.

QA methods have been developed to achieve submillimetric accuracy for stereotactic linear accelerators, as for example reported by Grimm et al. [8], mostly based on the Winston-Lutz test, using commercially available phantom and gafchromic films.

QA programs using the aSi-EPID images have been developed for both flattened [9] and unflattened beams [10].

Another example of QA program of a linear accelerator, specifically a Varian Unique machine, deeply using the imaging devices of the Linac has been published by Clivio et al. [11].

Specific regulations and recommendations are generally available in each country, with their own tolerance values and frequency of the specific checks. For example in Switzerland, as in many other countries all over the world, the Recommendations n. 11 [12] requires a quite labor intensive program, where the physicist has the faculty to implement different checks once judged appropriate, even if different from the suggested specific tests. This concept opens the possibility to implement in the routine checks new comprehensive programs that, with fast and reliable procedure, give many results of different sections of the entire QA program.

The Varian TrueBeam Linacs (Varian Medical Systems, Inc., Palo Alto, CA) are already equipped with a dedicated phantom and associated software, the IsoCal, an automated geometric calibration system for on-board imaging and MV imaging systems. Characteristics of the IsoCal are well described in the Gao et al. publication [13], where the authors applied the IsoCal calibration method on the Varian Clinac machines.

A step forward is now made available from Varian for TrueBeam platform version 2.0. It is the Machine Performance Check (MPC), an application and process to verify that Linac geometry and beam performances are operating within system specifications. This is done through automated checks based on the kV and MV imaging systems mounted on the Linac.

In the present study, preliminary tests with MPC were analyzed using all photon beam energies available on our TrueBeam. For each item analysed by the MPC, whenever possible, tests across the same time period using our routine procedures and detectors were also evaluated, as external independent checks for results comparison.

## Methods

**Figure 1:**
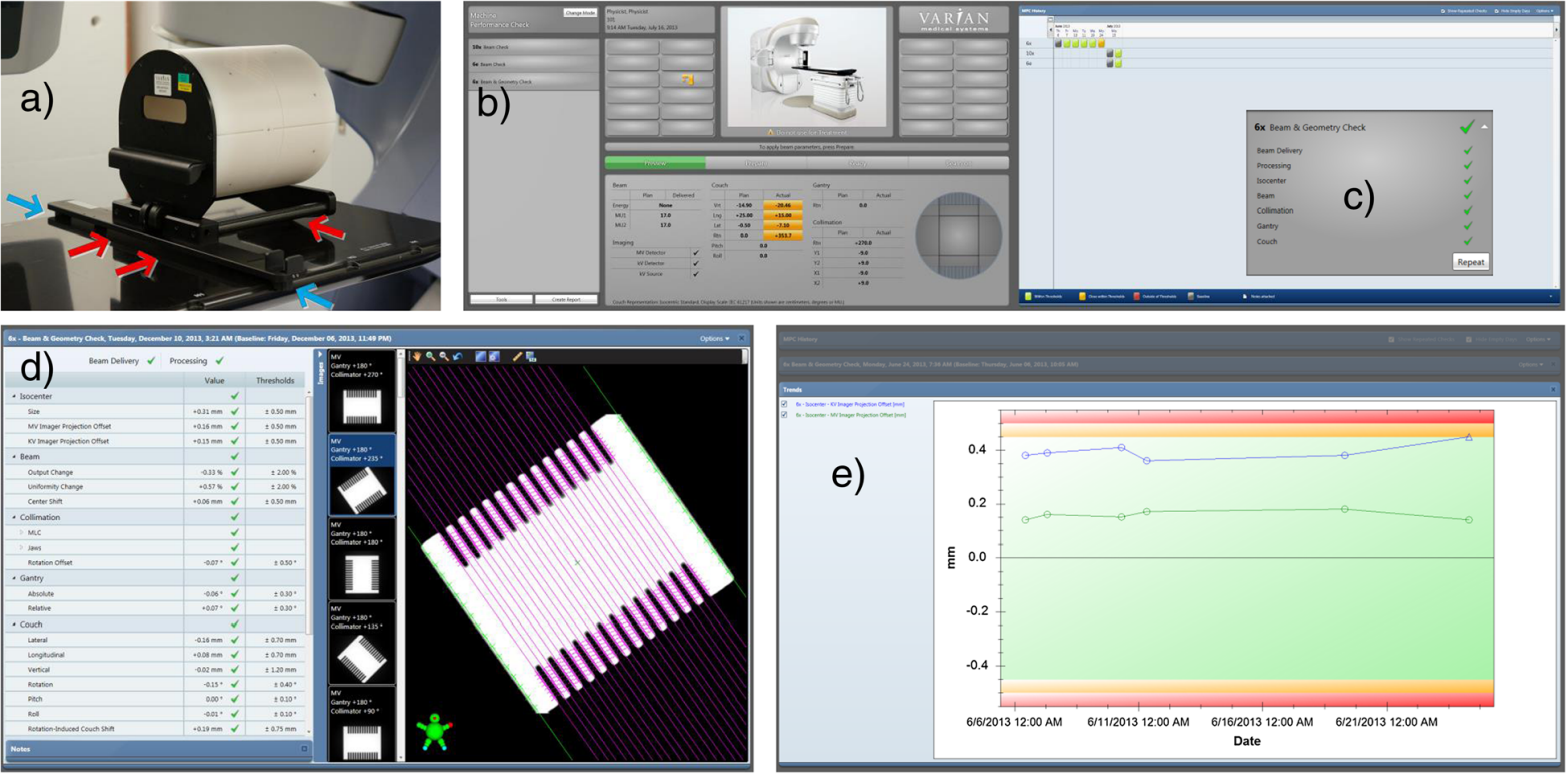
MPC components: **(a)** the IsoCal phantom mounted on the couch top, **(b)** the user interface, **(c)** the quick summary at the end of the MPC acquisitions **(d)** offline review of the parameters and images, **(e)** offline statistics of selected parameters.

The Machine Performance Check (MPC) is a new TrueBeam major mode, designed to evaluate the machines geometric performance in five minutes. It employs a fully automated measurement sequence that uses the kV and MV imaging systems and the proven IsoCal phantom [13]. The IsoCal phantom is a hollow cylinder 23 cm in diameter and length with 16 tungsten-carbide bearing balls (each 4 mm in diameter). For imager system calibration it uses a collimator plate attached to an accessory slot, having a steel pin in its center. For MPC implementation, the IsoCal phantom does not use such a collimator plate, and it is mounted to the couch top using a dedicated holder (Figure 1a). The operator enters into the MPC mode at the TrueBeam console (Figure 1b), and just initiates the procedure that takes place automatically. MPC automatically acquires a series of MV and kV images, moving the machine and imaging systems in the pre-defined positions. The two detector panels are positioned at a distance of 150 cm from the source. The images are immediately processed and the results displayed for a quick evaluation, indicating whether the values are within system specifications (Figure 1c). The results can also be reviewed offline (Figure 1d-e), exported and reported.

For the present study, a pre-released MPC version was used, and the sequences were run using the Varian Research Beam functionality.

The predefined acquisitions consist of a set of 39 images, 12 acquired with kV (XI system) with the IsoCal phantom in the field, 27 with MV (detected on the Portal Vision system), of which 20 are with and 7 without the IsoCal phantom in the field. The 39 images are the input for the machine performance parameter evaluation.

MPC has been here evaluated for the five photon energies available on our TrueBeam machine: 6, 10, and 15 MV and 6, 10 MV FFF.

For most of the MPC checks, an independent control has been performed at the same time of the acquisition of the MPC to evaluate the agreement of the two methods. For the independent checks, the here used procedures, phantoms and detectors were those available in the department and routinely used for quality assurance. They are not intended to be one-by-one tests relative to the MPC, but want to compare and discuss two different methodologies for checking the Linac performances.

First the MPC and then the independent checks are described below.

All data, MPC and independent checks, were acquired for 10 repetitions along a period of 3 weeks for the flattened beams, and a subsequent period of 3 weeks for the unflattened beams.

### MPC acquisitions

#### Geometry checks

The geometry checks evaluate the positioning accuracy of the various mechanical axes of the TrueBeam system. An important characteristic of the radiotherapy machine is the position and size of the treatment isocenter. For the MV and kV imager systems the important characteristic is the offset of the imager center relative to the treatment isocenter projection.

### Isocenter

The treatment isocenter is determined in MPC using the IsoCal phantom, which is located inside the beam during the acquisitions. It is defined as the ideal intersection point of the beam central axes over a full gantry rotation.

**Figure 2:**
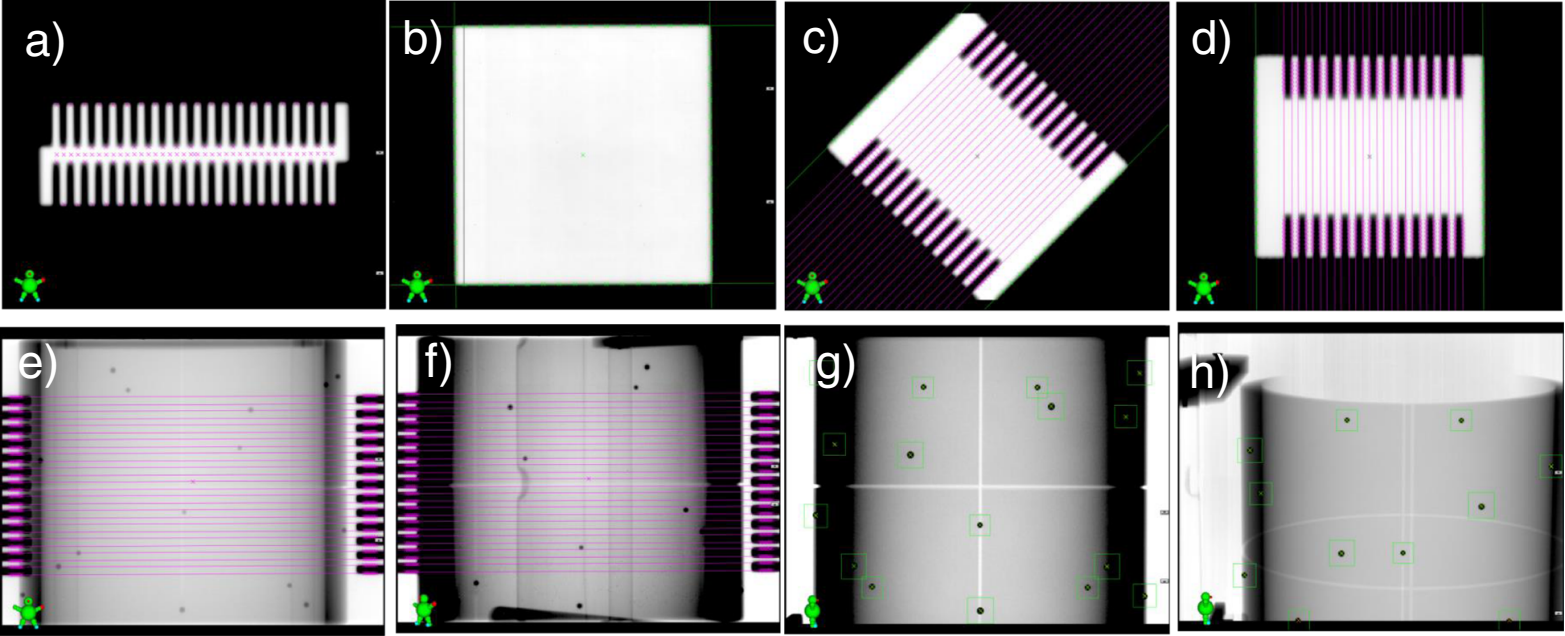
Examples among the 39 images needed for MPC evaluation. **(a)** comb-like pattern for MLC positioning (MV); **(b)** open beam for beam constancy (MV); **(c)** and **(d)** open field with comb-like MLC pattern for treatment isocenter definition, at two collimator angles (MV); **(e)** and **(f)** comb-like MLC pattern with IsoCal phantom for treatment isocenter and gantry position, at different gantry angles (MV); **(g)** and **(h)** IsoCal phantom with different couch positions (kV).

The beam central axis in MPC is defined, for each gantry angle and no IsoCal phantom in place, by the center of rotation of the MLC (considered as the highest priority collimating device), for five collimator rotations, 270°, 315°, 0°, 45°, 90° (Figure 2c and d: collimator rotation 45° and 90°), according to the procedure described in Additional file 1, Center of Rotation. The center of rotation of the MLC is determined with the edge detection of the MLC leaves (see Additional file 1, Edge Detection), positioned with a comb-like pattern at alternating 4 and 7 cm from the center line.

The treatment isocenter is then determined using acquisitions with the IsoCal phantom on eight gantry angles (0°, 45°, 90°, 135°, 180°, 225°, 270°, 315°), representative for the full gantry rotation (Figure 2e and f: gantry angles 0° and 45°). The parameters are determined from the position of the central beam axis and the expected positions of the 16 tungsten BBs of the phantom.

- **Size:** The size of the treatment isocenter is defined as the maximum distance of a beam central axis from the idealized isocenter.
- **MV and kV Imager Offset:** The imager projection offset represents the maximum distance of the imager center (MV and kV separately) from the projection of the treatment isocenter. It is a measure of the correctness of the IsoCal calibration.

### Collimation

The positioning accuracy of the whole collimation system is determined through static fields at gantry position 0°. The position of the collimating devices is evaluated in the acquired images as the point presenting the steepest gradient on a line profile perpendicular to the collimation edge. The in-plane rotation of the detector planes are measured as part of the gantry rotation shots. MPC can therefore compensate the collimator angle measurement for that rotation.

- **MLC (maximal offset, mean offset, individual offset, bank A/B)**
  The positioning accuracy of each MLC leaf is determined using a static comb-like pattern with alternating leaves. It is measured as the distance of the MLC leaf tip from the MLC center line (Figure 2a: the crosses represent the leaf tips and the corresponding points onto the MLC center line for calculating the distances). The center line is defined as the line through the center of rotation of the MLC that is perpendicular to the average leaf edges at the side (see Additional file 1 – Edge Detection, for the leaf edge evaluation). The average and maximum MLC offset, defined as the difference from the measured leaf tip position and the nominal value, is evaluated per each of the two banks. The MLC offsets are evaluated for a number of leaf pairs derived by the maximum field size detectable at a distance of 150 cm. For the Millennium-120 MLC used in the current testing phase, the central 40 leaf pairs (5 mm width) are evaluated.
- **Jaws (Offset X**_**1**_**/*X***_**2**_**/Y**_**1**_**/Y**_**2**_**)**
  Jaw edges are detected on a symmetric 18×18 cm^2^ field Figure 2b: the crosses represent the edge detection of the jaw setting, and the lines, fitting the edge detection, are the jaw edges. The central cross is the center of rotation of the MLC. The result is measured as the distance of the jaw edges from the center of rotation of the MLC. The rationale for the 18×18 cm^2^ field size choice is related to the aS1000 imager dimension (40×30 cm^2^) and position (distance of 150 cm from the beam source): the field size scales by 1.5, leading to using the 18×18 cm^2^ (27 cm side at the imager level) as the largest field size that allows a reliable field edge detection.
- **Rotation Offset**
  The rotation offset is determined as the maximum deviation of the measured collimator angle, as defined by the leaf edges, versus the nominal one.

### Gantry

The MPC geometry check evaluates two characteristics of the machines gantry positioning system, absolute and relative:

- **Absolute**
  The absolute positioning accuracy is defined as the coincidence of the couch vertical axis with the central beam axis at gantry 0°, evaluated with MV and kV images with the IsoCal phantom and the couch at different heights. MPC evaluates any lateral shift of the phantom with respect to the beam and the treatment isocenter as the absolute gantry angle positioning error. See Additional file 1, Gantry Absolute Positioning for more details on the procedure.
- **Relative**
  The relative positioning accuracy of the gantry is the maximum deviation between the actual angle determined with the MV images with the IsoCal phantom, and the nominal gantry angle. The values are compared for eight representative gantry angles (0, 45, 90, 135, 180, 225, 270, 315°).

### Couch

MPC measures the positioning accuracy of the different couch axes with respect to a reference position (established as the fixed room coordinate system using MV and kV images with the IsoCal phantom). Subsequently, the couch axes are moved and the actual distances are determined.

- **Lateral:** describes the positioning accuracy of the lateral couch axis on a 5 cm travel range.
- **Longitudinal:** describes the positioning accuracy of the longitudinal couch axis on a 5 cm travel range.
- **Vertical:** describes the positioning accuracy of the vertical couch axis on a 15 cm travel range.
- **Rotation:** describes the positioning accuracy of the patient support angle on a 10° travel range.
- **Pitch and Roll:** describes the positioning accuracy of the patient pitch and roll angles on a 3° travel range (only for PerfectPitch couch top, not evaluated in the current study).
- **Rotation-Induced Couch Shift:** describes the distance between the center of rotation of the couch, determined through a motion on the rotational axes, and the treatment isocenter.

### Baseline

MPC does not use any external equipment for measuring dosimetric properties of the beam, but it is based on the concept of baseline data. A reference state of the machine is marked as baseline, with which subsequent acquisitions are compared to. Being a relative evaluation in its nature, a baseline acquisition has to precede any check. A baseline should be acquired only when the dosimetric performance of the beam is verified by independent means (e.g. ion chamber measurements). The baselines used in the current work refer to the first acquisition with MPC, prior to the 10 repetitions.

### Beam constancy checks

To evaluate the beam constancy, MPC uses an uncorrected MV portal image (i.e. not corrected for the flood field) of a symmetric, jaw-collimated (18×18 cm^2^) field at gantry 0°. Ratio images are calculated between the baseline and the image of the checking beam for each energy. To reduce the impact of the jaw positioning, the following parameters are evaluated on a central area of 13.3×13.3 cm^2^ of the ratio image field.

### Beam output change

It represents the average percentage variation in detector response as mean of the ratio between the beam check acquisition and the baseline data, in the central area of the imager. For this evaluation, high frequency noise is filtered from the ratio image.

### Beam uniformity change

It represents the percentage variation of the uniformity between the current and the baseline image. The uniformity is defined as the difference between the two pixels with the lowest and the highest ratio in the central area of the imager. It is not an evaluation of the beam symmetry. For this evaluation, high frequency noise is filtered from the ratio image.

### Beam center shift

It describes the relative shift of the field center, defined by a jaw-collimated field, with respect to the baseline. The field center is found through detection of the jaw edges in the beam image. This shift accounts for the precision of the beam steering system, the collimation and the MV imaging system.

### Independent checks

#### Geometry checks

The routine checks, according to internal protocols, were performed to evaluate the different geometry tests.

### Isocenter

**Figure 3:**
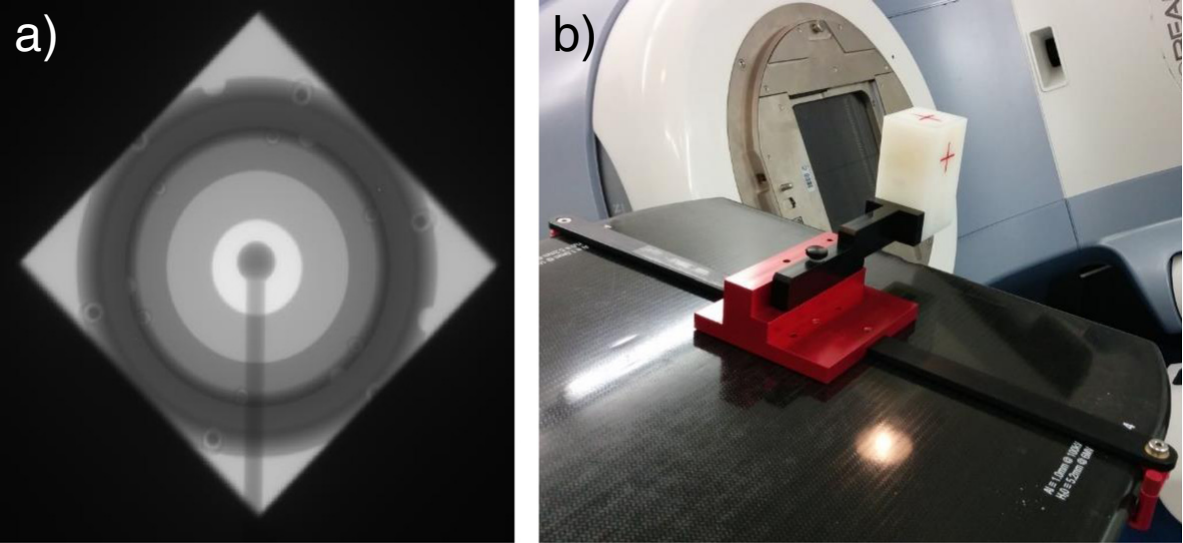
Set-up of treatment isocenter tests. **(a)** A field acquired by the IsoLock for the Winston-Lutz test; **(b)** the MarkerBlock phantom for the two orthogonal images acquisition.

The treatment isocenter was evaluated with a complete Winston-Lutz test [14] for each energy, using the procedure, software and toolkit provided by Varian and commonly used during machine installations (named IsoLock). In Figure 3a a field acquired by the IsoLock, showing the set-up for the Winston-Lutz test.

The imagers isocenter (kV and MV together) was checked using the MarkerBlock phantom provided by Varian (Figure 3b), by acquiring two orthogonal MV and kV images. The phantom contains a small, well-defined radio-opaque structure that has to be positioned at isocenter; isocenter location is verified by matching the two orthogonal images.

### Collimation

To evaluate MLC leaf positioning the picket fence test was evaluated. The test pattern was acquired with the portal imager, where discrepancies ≥0.5 mm can be visually detected thanks to the color scales, as proven with the same test pattern delivered with intentional errors of 0.5 mm.

To evaluate the jaw position the PTW StarCheck acquisitions were used for the 10×10 and 20×20 cm^2^ fields: jaw position was determined according to the field size calculated by the Mephysto software (PTW, Freiburg, Germany). A pre-release version of Mephysto has been used to evaluate the field size according to the re-normalization of unflattened beam profiles, according to Fogliata et al. [5], to have a check which is compatible between flattened and unflattened beams. Only the 20×20 cm^2^ field results are here reported, as more consistent with the 18×18 cm^2^ field size set for MPC checks. The PTW StarCheck is a 2-D array of 527 vented ion chambers with a volume of 0.08 cm^3^, aligned along the two main axes and the two diagonals with 3 mm spatial resolution; a dedicated software analyzes the most important parameters of the beam profiles. Detector was aligned according to the cross-hair (light field). Such a positioning has an intrinsic accuracy (given by the shadow visibility) that could be estimated as 0.5 mm. To question is the choice of the alignment respect to the cross-hair instead of the light field edges: if the cross-hair is misplaced relative to the beam center, the single jaw position check is then affected by that uncertainty, that could could reach 1 mm.

For the collimator rotation a spirit level was used with the gantry at 90° and 270° (averaging the positions that might differ due to gantry sag), and the collimator set to 0, 90, 270°.

### Gantry

For the mechanical checks an analog spirit level was used to compare to the digital reading at gantry positions of 0, 90, 270 and 180°.

### Couch

The test was performed by moving the couch by 10 cm in the 6 available directions (±lateral, ±longitudinal, and ± vertical) and recording the shifts relative to the actual movement read on a millimetric paper or ruler. For rotation, the couch was rotated according to the orthogonal lines on the paper (couch at 0, 90, 270°), and the difference relative to the digital readout was recorded.

### Beam constancy checks

The PTW StarCheck has been used to independently check the beam constancy. A 10×10 and a 20×20 cm^2^ field were acquired with the detectors at isocenter distance. The first acquisition, acquired at the same time as the MPC baseline, was used as reference.

### Beam output

The central ion chamber reading, corrected for pressure and temperature, was compared against the reference.

### Beam uniformity

The percentage difference of the (D_max_-D_min_)/2 values along the two main axes was referred to the same values from the reference.

## Results and discussion

Some examples of the acquired images are shown in Figure 2, with kV and MV maps, with and without the IsoCal in the beam, and the comb-like pattern, according to the descriptions in the Methods section.

For each parameter a threshold value is used by the MPC software that represents the corresponding TrueBeam system specification, and is reported in Tables 1 and 2, together with the measured parameter. Values exceeding their threshold (or are near to it) is highlighted by the MPC software in red (or yellow) to warn the user (Figure 1c). For the acquisitions of the present study only the 6MV FFF results of three repetitions were highlighted in red, and will be discussed later. All other cases passed the checks. The same threshold values were considered also for the independent checks, since they correspond to the machine specifications.

**Table 1:** **Geometric checks: MPC and independent checks for all evaluated parameters, all energies**

**Parameter**	**Check**	**Thresh**	**6MV**	**10MV**	**15MV**	**6MV FFF**	**10MV FFF**
**ISOCENTER**							
Isocenter size [mm]	MPC	±0.50	0.34 ± 0.01	0.31 ± 0.01	0.34 ± 0.01	0.42 ± 0.02	0.37 ± 0.02
*Winston-Lutz [mm]*	*Indep*		*0.264*	*0.271*	*0.257*	*0.289*	*0.278*
MV imager proj. offset [mm]	MPC	±0.50	0.17 ± 0.03	0.17 ± 0.04	0.29 ± 0.03	0.43 ± 0.06	0.15 ± 0.04
*MV imager proj. offset [mm]*	*Indep*		*0.3 ± 0.2*	*0.3 ± 0.2*	*0.3 ± 0.2*	*0.3 ± 0.1*	*0.3 ± 0.1*
kV imager proj. offset [mm]	MPC	±0.50	0.32 ± 0.02	0.33 ± 0.03	0.29 ± 0.03	0.29 ± 0.04	0.36 ± 0.03
*kV imager proj. offset [mm]*	*Indep*		*0.4 ± 0.1*	*0.4 ± 0.1*	*0.4 ± 0.1*	*0.3 ± 0.2*	*0.3 ± 0.2*
**COLLIMATION**							
MLC: Max Offset A [mm]	MPC	±1.00	−0.35 ± 0.03	−0.40 ± 0.03	−0.38 ± 0.02	−0.26 ± 0.02	−0.35 ± 0.02
MLC: Max Offset B [mm]	MPC	±1.00	0.44 ± 0.02	0.56 ± 0.01	0.54 ± 0.01	0.34 ± 0.02	0.45 ± 0.02
MLC: Mean Offset A [mm]	MPC	±1.00	−0.24 ± 0.02	−0.30 ± 0.02	−0.27 ± 0.02	−0.16 ± 0.02	−0.25 ± 0.02
MLC: Mean Offset B [mm]	MPC	±1.00	0.25 ± 0.02	0.36 ± 0.01	0.34 ± 0.01	0.15 ± 0.02	0.26 ± 0.01
*MLC: Max Offset A [mm]*	*Indep*	*±1.00*	*≤0.5 ± 0.5*	*≤0.5 ± 0.5*	*≤0.5 ± 0.5*	*≤0.5 ± 0.5*	*≤0.5 ± 0.5*
*MLC: Max Offset B [mm]*	*Indep*	*±1.00*	*≤0.5 ± 0.5*	*≤0.5 ± 0.5*	*≤0.5 ± 0.5*	*≤0.5 ± 0.5*	*≤0.5 ± 0.5*
Jaws: Offset X1 [mm]	MPC	±1.00	0.01 ± 0.01	0.04 ± 0.01	0.04 ± 0.01	0.04 ± 0.02	0.06 ± 0.01
Jaws: Offset *X*2 [mm]	MPC	±1.00	0.06 ± 0.02	0.13 ± 0.02	0.11 ± 0.02	0.06 ± 0.03	0.16 ± 0.02
Jaws: Offset Y1 [mm]	MPC	±2.00	−1.02 ± 0.05	−0.98 ± 0.01	−0.98 ± 0.01	−1.06 ± 0.03	−1.00 ± 0.01
Jaws: Offset Y2 [mm]	MPC	±2.00	0.87 ± 0.04	0.95 ± 0.03	0.90 ± 0.03	0.89 ± 0.03	0.98 ± 0.01
*Jaws: Offset X1 [mm]*	*Indep*		*0.6 ± 0.2*	*0.9 ± 0.2*	*0.8 ± 0.2*	*0.2 ± 0.3*	*0.5 ± 0.3*
*Jaws: Offset X2 [mm]*	*Indep*		*0.8 ± 0.2*	*0.9 ± 0.2*	*1.2 ± 0.2*	*1.0 ± 0.3*	*1.1 ± 0.3*
*Jaws: Offset Y1 [mm]*	*Indep*		*−0.1 ± 0.2*	*0.3 ± 0.2*	*0.3 ± 0.2*	*−0.6 ± 0.3*	*0.2 ± 0.2*
*Jaws: Offset Y2 [mm]*	*Indep*		*−0.1 ± 0.3*	*0.1 ± 0.2*	*0.3 ± 0.2*	*0.1 ± 0.2*	*−0.1 ± 0.2*
Collimator rotation offset [°]	MPC	±0.50	0.17 ± 0.01	0.16 ± 0.01	0.16 ± 0.01	0.16 ± 0.01	0.16 ± 0.01
*Collimator rotation offset [°]*	*Indep*		*−0.1 ± 0.1*	*−0.1 ± 0.1*	*−0.1 ± 0.1*	*−0.1 ± 0.1*	*−0.1 ± 0.1*
**GANTRY**							
Absolute [°]	MPC	±0.30	−0.09 ± 0.02	−0.09 ± 0.02	−0.10 ± 0.03	−0.10 ± 0.01	−0.09 ± 0.02
Relative [°]	MPC	±0.30	0.08 ± 0.01	0.09 ± 0.01	0.09 ± 0.01	0.09 ± 0.01	0.09 ± 0.01
*Relative [°]*	*Indep*		*0.0 ± 0.1*	*0.0 ± 0.1*	*0.0 ± 0.1*	*0.0 ± 0.1*	*0.0 ± 0.1*
**COUCH**							
Lateral [mm]	MPC	±0.70	−0.06 ± 0.02	−0.07 ± 0.02	−0.05 ± 0.02	−0.04 ± 0.02	−0.07 ± 0.02
Longitudinal [mm]	MPC	±0.70	0.14 ± 0.02	0.12 ± 0.01	0.14 ± 0.02	0.20 ± 0.04	0.13 ± 0.02
Vertical [mm]	MPC	±1.20	−0.34 ± 0.01	−0.33 ± 0.02	−0.32 ± 0.01	−0.32 ± 0.02	−0.33 ± 0.02
Rotation [°]	MPC	±0.40	0.00 ± 0.01	0.00 ± 0.01	0.00 ± 0.01	0.01 ± 0.01	0.00 ± 0.01
Rotation-induced shift [mm]	MPC	±0.75	0.37 ± 0.02	0.36 ± 0.01	0.35 ± 0.02	0.37 ± 0.02	0.38 ± 0.02
*Lateral [mm]*	*Indep*		*0.1 ± 0.2*	*0.1 ± 0.2*	*0.1 ± 0.2*	*0.1 ± 0.2*	*0.1 ± 0.2*
*Longitudinal [mm]*	*Indep*		*0.3 ± 0.4*	*0.3 ± 0.4*	*0.3 ± 0.4*	*0.3 ± 0.4*	*0.3 ± 0.4*
*Vertical [mm]*	*Indep*		*−0.1 ± 0.3*	*−0.1 ± 0.3*	*−0.1 ± 0.3*	*−0.1 ± 0.1*	*−0.1 ± 0.1*
*Rotation [°]*	*Indep*		*0.0 ± 0.1*	*0.0 ± 0.1*	*0.0 ± 0.1*	*0.0 ± 0.1*	*0.0 ± 0.1*
*Winston-Lutz [mm]*	*Indep*		*0.803*	*n/a*	*n/a*	*n/a*	*n/a*

**Table 2:** **Dosimetric constancy checks: MPC and independent checks for all evaluated parameters, all energies**

**Parameter**	**Check**	**Thresh**	**6MV**	**10MV**	**15MV**	**6MV FFF**	**10MV FFF**
**BEAM**							
Output Change [%]	MPC	±2.0	0.15 ± 0.13	0.10 ± 0.29	0.06 ± 0.15	0.24 ± 0.24	0.22 ± 0.16
*Output Change [%]*	*Indep*		*0.4 ± 0.4*	*0.4 ± 0.5*	*0.3 ± 0.6*	*−0.1 ± 0.4*	*0.5 ± 0.3*
Uniformity Change [%]	MPC	±2.0	0.77 ± 0.20	0.92 ± 0.13	0.94 ± 0.21	0.88 ± 0.19	0.89 ± 0.12
*Uniformity Change [%]*	*Indep*		*−0.1 ± 0.1*	*−0.2 ± 0.2*	*0.4 ± 0.3*	*0.1 ± 0.1*	*0.1 ± 0.1*
Center Shift [mm]	MPC	±0.5	0.04 ± 0.02	0.04 ± 0.01	0.04 ± 0.01	0.15 ± 0.02	0.09 ± 0.02

### Evaluation accuracy

The accuracy of MPC evaluated parameters includes data acquisition (measurement accuracy) as well as image processing (algorithm accuracy). The measurement accuracy is the ceil-rounded standard deviation for each value based on data from a 6 weeks period of daily measurements. The algorithm accuracy is estimated using synthetic data. Datasets with varying parameters (e.g. shifted phantom, rotated imager) were generated. The mean + 10 times the standard deviation for each value is rounded to the next higher decimal to give the algorithm accuracy for each value. In Table 3 are reported both the algorithm and measurement accuracies.

**Table 3:** **MPC accuracy for all evaluated parameters**

**MPC performance value**	**Algorithm accuracy**	**Measurement accuracy**
Beam – Output change [%]	-	0.3
Beam – uniformity change [%]	-	0.2
Beam – center shift [mm]	-	0.04
Isocenter size [mm]	±0.01	0.01
MV imager projection offset [mm]	±0.02	0.03
kV imager projection offset [mm]	±0.1	0.1
MLC – maximal offset leaves A/B [mm]	±0.1	0.1
MLC – mean offset leaves A/B [mm]	±0.02	0.05
Jaws – offset X1/*X*2/Y1/Y2 [mm]	±0.05	0.07
Collimator rotation offset [°]	±0.005	0.008
Gantry – absolute [°]	±0.01	0.01
Gantry – relative [°]	±0.02	0.02
Couch – lateral/longitudinal/vertical [mm]	±0.01	0.04
Couch – rotation/pitch/roll [°]	±0.01	0.01
Couch – rotation-induced couch shift [mm]	±0.01	0.03

The accuracy of the independent measurements was mostly due to the instrument precision for the geometric checks, i.e. 0.5 mm for the linear couch movements, 1 mm for the collimation settings, 0.5° for the rotational movements. The measurement accuracy was here reported as standard deviation of the repeated tests. For beam constancy checks the accuracy was evaluated within 0.5%.

### Geometric checks

Geometric checks data are reported in Table 1, as the mean values over the 11 acquisitions (10 repetitions and the baseline); the uncertainty is expressed as one standard deviation on all the acquisitions.

For one acquisition session (including all flattened beams), all the couch and gantry group values were unavailable, as the necessary markers in the IsoCal phantom could not be detected, as they followed out of the expected area, due to a wrong placement of the IsoCal phantom on its holder for that measurement session, with a shift of more than 5 mm relative to the correct position. The phantom misplacement was then corrected by the software, and the results were consequently adjusted. For the subsequent released version of MPC (the version used in the present study was a pre-released version), a misplaced phantom won’t influence any measured value as long as all the markers and features could be detected. If this won’t be the case, the overall check results will be shown as “failed”, meaning that MPC cannot detect all features.

As expected, from Table 1 there are no considerable differences among the energies in the average values of the geometric parameters. In particular, we should expect practically identical data for the kV image related checks, as long as kV imager is not influenced by the treatment beam. The kV imager checks are of primary importance, as the kV imager isocenter (offset) is the guarantee of an accurate matching of all patient positioning, 2D or Cone-Beam-CT image based.

Coincidence of MV and kV imaging isocenters with respect to the treatment isocenter position resulted well consistent between MPC and independent check. Also the isocenter size showed to be well consistent between MPC and the Winston-Lutz test (with the largest difference of less than 0.15 mm for 6FFF). The collimator rotation, gantry and couch positions are well in agreement between MPC and routine checks. The Winston-Lutz test with the couch rotation performed for 6 MV only showed a shift of 0.8 mm, to compare with the rotation-induced couch shift of 0.4 mm evaluated by MPC.

**Figure 4:**
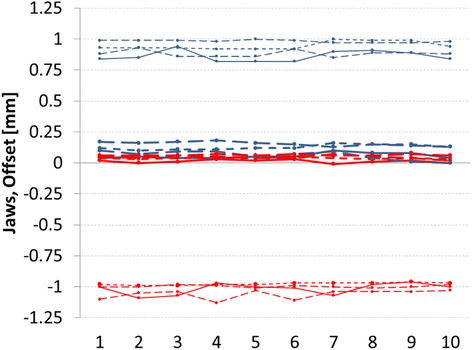
Jaw Offset for all repetitions. Red lines refer to X1 and Y1 jaws, blue lines to *X*2 and Y2 jaws. Thick lines refer to X1 and *X*2 jaws, thin lines to Y1 and Y2 jaws. The different line styles belong to different energies (6 and 10 MV, 6 and 10 MV FFF).

From all MPC acquisitions the Y jaws parameters showed an offset for Y1 of about −1 mm for all energies, and +0.9 mm for Y2. This suggested an inaccurate calibration of the two Y jaws, probably due to a misalignment of the cross-hair. To note that the independent checks did not confirm such offsets; possible concurrent causes could be the fact that the StarCheck was centered with respect to the cross-hair (not to the treatment beam center), and the 3 mm resolution of the detector that could be not enough to detect small discrepancies. An additional check of the cross-hair position with the collimator rotation showed a misalignment of the cross-hair of 0.5-1 mm, as measurable with a shadow of a line on a piece of paper. With the MPC it was on the contrary possible to clearly and precisely detect such an inaccuracy in the jaw calibration, that was then corrected. The jaw setting resulted consistent for all energies and during the whole evaluation period, confirming the possible problem in jaw calibration. In Figure 4, as an example, the values of the jaw parameters are shown.

Independent checks for multileaf collimator positioning showed, in the analysed period, no detectable deviations, that means shifts not larger than 0.5 mm. MPC confirmed the accurate leaf positioning and its constancy (shown as small standard deviation).

A limitation of the MPC is the fact that the MLC and jaw offsets are evaluated for single positions, i.e. a single comb-like pattern for MLC and a 18×18 cm^2^ for jaws. It would be advisable to add in the MPC programme at least another MLC pattern and another field size, exploring a wider range of distances to include a check of linearity of the leaf and jaw positioning, possibly on the widest possible range. The same missing linearity test in MPC is on most of the geometry checks, where a single shift or rotation is evaluated. The inclusion in the MPC programme of the linearity concept, adding at least another test point in all geometry checks (possibly covering the widest possible range) would add completeness to the MPC tests as comprehensive machine performance check package.

### Beam consistency checks

Dosimetric data are reported in Table 2 as the mean values of the 10 repetitions; the uncertainties are expressed as one standard deviation.

For dosimetric checks one case presented the Output Change value out of the threshold, of −2.86% (value not included in the statistics of Table 2 for the reason explained here below) for the 6 MV FFF beam. For the same 6 MV FFF beams, for three consecutive MPC checks (one of those was the same session presenting the bad output) showed an Uniformity Change parameter out of tolerance. In those cases the images presented a saturation effect. In the tested pre-release version of MPC, the beam checks could encounter imager saturation with 6 MV FFF energy. This is due to the relatively higher detector sensitivity to low energy photons: the detector counts for unflattened (lower energy than flattened) and low energy beams would approach the saturation limit for the imager electronics. This issue has been solved in the subsequent released version, where the acquisition procedure has been improved: for each energy the acquisition procedure was changed to have optimal response characteristics from the imager, with different number of frames and dose per frame. The results affected by the saturation problems in this study (having used the pre-released version) were not included in the analysis summarized in Table 2.

The independent checks with the StarCheck presented stable beams both in output and uniformity.

A limitation of the MPC beam consistency check relates to the analysis of the beam uniformity, that refers to the whole field area, while common tests and parameters are based on the main axis profiles. In particular, flatness (or unflatness in case of FFF beams) and symmetry are often used to evaluate a beam. A single uniformity value on the whole area might hide potential problems in evaluating this common aspect. It could therefore be advisable to include in the MPC programme also an analysis on the main axis profiles extracted by the whole MV image.

The MPC is an approach for a comprehensive performance check against machine specifications of the whole TrueBeam system, including the imaging system of the treatment beam. The time required for the acquisition of all the 39 required images was on average 5.6 ± 0.5 min per energy. The whole process of the automization to acquire all the images in a single plan makes MPC a very fast approach, being the machine occupancy of about 30 minutes to check all the five energies of TrueBeam, even including the time for setting up the IsoCal phantom on its holder. In comparison, the normal routine checks generally are spread over a number of different tests on the specific items, using different phantoms, detectors and set-up, needing much longer time, that could easily last hours, to acquire all the tests on the machine.

Evaluating the results compared to independent checks, the MPC could be easily and safely used for even daily checks, leaving a deeper quality assurance control for a lower frequency, especially for what concerns the dosimetric QA program, also depending on the specific country regulations. In particular, as an example, the majority of the requests of the Swiss regulations for Linac quality assurance [12] can be easily and fast satisfied with this tool.

Point of improvements have been discussed, and mostly relate to the possibility to add linearity checks.

Acquisitions and evaluations over a longer period would allow to better understand the stability and full reliability of the dosimetric checks. To consider in any case that the beam constancy checks cannot be considered as true dosimetric quality assurance controls, being only relative to the baseline MV images. MPC is not indeed intended a machine QA tool and does not replace the need to perform routine QA. Varian recommends that institutions follow accepted QA guidelines.

A limitation of the current study is the introduction at the machine level of known errors on purpose (changing the output, or the beam symmetry, or the jaw setting to cite some of the tests), in order to quantify the sensibility of the MPC parameters related to the known errors. This is the subject for the next study.

## Conclusions

MPC proved to be a reliable, fast and easy to use method for checking the machine performances on both geometric and dosimetric aspects and showed to be in agreement with the checks performed with a more conventional approach.

## Additional file

Additional file 1:
**MPC acquisition procedures for Center of Rotation, Edge Detection, and Gantry Absolute Positioning.**
