# Supplementary material for: Evaluation of the Machine Performance Check application for TrueBeam Linac
Source: Radiat Oncol. 2015 Apr 21;10:97. doi: 10.1186/s13014-015-0381-0 (PMC4464869; doi:10.1186/s13014-015-0381-0)
Supplement: Additional file 1: — MPC acquisition procedures for Center of Rotation, Edge Detection, and Gantry Absolute Positioning. [file 13014_2015_381_MOESM1_ESM.docx]

**Additional file 1**


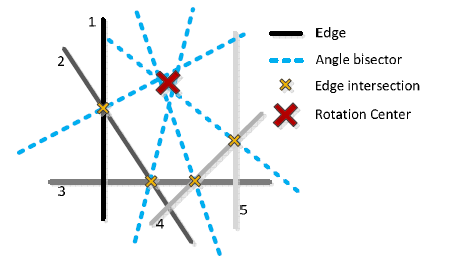


**Center of Rotation**. It is the center of rotation of the collimating devices, calculated from the detected edges for the five collimator rotation shots. For each pair of subsequent edges, the angle bisector line is calculated, resulting in four angle bisectors. A least squares finds the point in space with the least distance to all bisection lines.


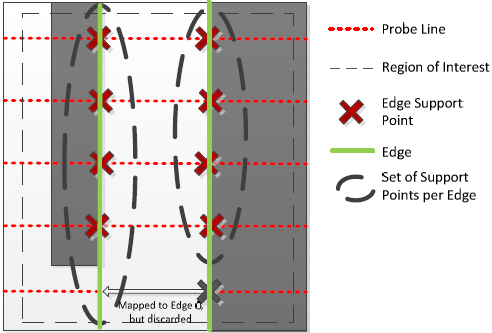


**Edge detection**. A set of probe lines perpendicular to the edge to detect is defined in a region of interest. Each of the probe is filtered for spanned pixel information (Savitzky-Golay filter). Along these filtered lines, inflection points are found (Edge Support Points). A line is fit according to the valid inflection points for each edge. Erroneous points are detected and removed from the fit.

**Gantry Absolute Positioning**. The beam center axis shall coincide with the couch vertical axis for a gantry position of 0°. In a first step, the phantom is positioned at the isocenter. MV and kV images are acquired and the phantom position is fit in 6 degree of freedom with respect to the MV imaging system for establishing the reference position. In a second step, the couch is moved by a known distance in vertical direction. Again, MV and kV images are **
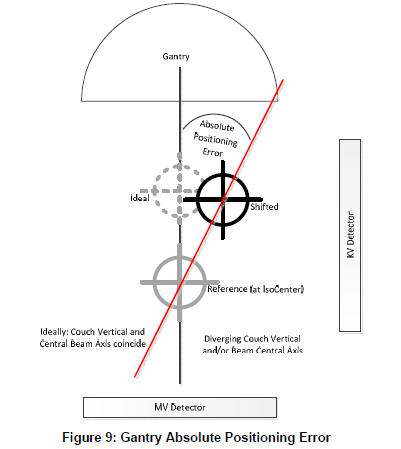
**acquired and the phantom position is again fit. In the ideal case, the transformation just contains a vertical component, yielding a zero angular error for the absolute gantry position. If this is not the case, the absolute positioning error is calculated as the arctan of the ratio of the X/Z component of the shift vector.
